# Prevalence and health correlates of workplace violence and discrimination against hospital employees – a cross-sectional study in German-speaking Switzerland

**DOI:** 10.1186/s12913-022-07602-5

**Published:** 2022-03-03

**Authors:** Alenka Stahl-Gugger, Oliver Hämmig

**Affiliations:** 1 Health Directorate of the Canton of Zurich, Stampfenbachstrasse 30, 8090 Zurich, Switzerland; 2 Epidemiology, Biostatistics and Prevention Institute of the University of Zurich, Hirschengraben 84, 8001 Zurich, Switzerland

**Keywords:** Workplace violence, Workplace discrimination, General health, Mental health, Hospital employees

## Abstract

**Background:**

Violence and discrimination are common events at work, especially in health care. Moreover, such workplace experiences are considered to have negative impacts and particularly adverse health consequences on health care workers. Nevertheless, the problem is still highly underreported and thus largely ignored and unexplored in Switzerland as comprehensive data and studies on their prevalence and health correlates among hospital staffs and health professionals are widely missing.

**Methods:**

This cross-sectional study was based on secondary data from a company survey among several public hospitals and rehabilitation clinics in German-speaking Switzerland conducted in 2015/16. The study population was limited to a subsample of 1567 health professionals among the surveyed staffs of five participating hospitals and clinics. Relative frequencies of different forms of violence and discrimination at work and the total number of such experiences were calculated for the entire study population and for occupational subgroups. These data were compared with a nationally representative subsample of the Swiss Health Survey 2017 as a reference population. Multiple logistic regression analyses were further computed to investigate the association between the number of different experienced forms of violence and/or discrimination at work and several poor general and mental health outcomes.

**Results:**

23% of the inverviewed hospital employees experienced at least one form of discrimination or violence at work in the past year, compared to 18% of the general working population. Nurses were by far the most affected occupational group regarding all forms of violence. More and particularly the most exposed and affected hospital employees with regard to experiences of violence and/or discrimination at work showed almost consistently increased frequencies and relative risks for the studied poor mental and general health outcomes. Prevalence rates and odds ratios for strong sleep disorders, strong stress feelings and increased burnout symptoms were between 3 and 4 times higher among the most exposed compared to the non-exposed group of hospital employees.

**Conclusions:**

Study findings underline the importance of an active combat against violent and discriminatory behaviors in health care. Prevention strategies should particularly focus on nurses and midwives, which turned out to be the most affected and exposed group of all health professions.

## Background

Workplace violence or aggression is not a new phenomenon but a serious and growing problem that can be found in virtually all occupations [1]. And workplace violence is not a marginal phenomenon but rather an everyday event that is frequently observed and highly prevalent in social services and particularly in health care settings and among nurses [2–5]. And although workplace violence against health care workers is increasingly recognized and addressed in health (services) research, in public, practice and politics it always was and still is strongly underreported and largely ignored or tolerated [6].

All this applies basically also to workplace discrimination which however does not seem to be generally and particularly widespread in the health care sector and among health professions compared to other industries and professions. However, workplace discrimination against health care workers and particularly based on gender, age, national origin or race/color is reported to be more common at least in individual countries such as Finland or the United States or among physicians and in specific settings or medical disciplines such as surgery [7–12].

Against the background of the current research literature, it seems that – within the health care sector and across countries – workplace violence most frequently occurs in nurses whereas workplace discrimination is most prevalent among physicians.

The International Labour Organization (ILO) of the United Nations defines workplace violence as “any action, incident or behavior that departs from reasonable conduct in which a person is assaulted, threatened, harmed, injured in the course of, or as a direct result of, his or her work” [13]. Discrimination on the other hand is defined by the ILO as “any distinction, exclusion or preference made on the basis of race, color, sex, religion, political opinion, national extraction or social origin, which has the effect of nullifying or impairing equality of opportunity and treatment in employment or occupation” [14].

There are many different forms of discrimination and violence. The latter can be divided into a physical, a psychological and a sexual dimension according to the ILO [13]. Additionally, a distinction between internal (between employees) and external violence (between an employee and another person present at work) can be made [1].

Workplace violence against health care workers seems to be most common in the forms of verbal abuse and sexual harassment and particularly widespread in North American and Asian countries [3, 4, 6, 15, 16]. According to several systematic reviews up to two thirds of the nurses and other health professionals are exposed to violence at the workplace within a year, depending on the profession, setting (or context), country or study [3–5, 15, 17, 18]. Health care workers are particularly affected from workplace violence and regularly confronted with aggression from patients or their relatives mainly because they are in frequent or even daily contact with people in distress and despair and/or with mental health problems [19]. Among all health professionals, nurses and – to a lesser extent and at a lower frequency – also physicians are especially vulnerable and most exposed to workplace violence and frequently experience aggression from patients or relatives [2, 5, 6, 15, 20]. Workplace violence in general and physical aggression in particular occur most frequently in psychiatric institutions, nursing homes and emergency departments [3, 6, 14, 17, 21, 22].

Discrimination and unfair treatment at the workplace is usually less prominent and prevalent than workplace violence and aggression but nevertheless present and quite common in health care settings and among hospital staffs and particularly physicians [7, 10, 11, 23]. However, strongly varying proportions up to 70% of the examined physicians in specific subgroups or specialties had experienced (or witnessed) and reported age, gender or racial discrimination during their medical careers depending very much on the age, gender, ethnic origin or country and study [7, 9–11].

In spite of such studies and systematic reviews on the general, global, national or setting-, gender- or profession-specific prevalence, the true extent of workplace violence and discrimination in the health care sector is difficult to assess, as a high number of unreported cases must be assumed [10, 20, 24]. Reported or assumed reasons for a systematic underreporting of experienced or witnessed workplace violence and discrimination are that employees do not anticipate a change or that they underestimate or are afraid of its negative consequences [10, 20, 25]. Underreporting of workplace violence against nurses or workplace discrimination against physicians is a particular problem in health care because of the direct patient contact or the power structures in hospital settings (unequal power relations between the health professions or sexes). Health professionals who are victims of violence or discrimination at the workplace tend to excuse or downplay the aggressive or unfair and discriminatory behavior of patients, relatives, colleagues or supervisors, for example with the health status of the patients [26] or due to existing structures or missing contact persons and responsible authorities [10].

Although workplace violence and discrimination are completely different phenomena, they have two things in common: They have been both observed as present and comparably prevalent in the health care sector or at least in specific health care settings and professions. They are both expected and found to be major health threats to those affected [27]. But in contrast to the prevalence, the consequences of workplace violence and discrimination against health care workers are less studied and evaluated. However, there is evidence from several studies that such negative experiences at work among clinical nurses and physicians or other health professionals are associated with specific health and work-related outcomes such as burnout, sleep problems, job dissatisfaction or turnover intention [3, 8, 10, 28]. A systematic review revealed a broad range of negative consequences of workplace violence in the health care setting [29], which can be divided into seven categories: physical consequences (direct injuries of the body), psychological consequences (as depressive or burnout symptoms), emotional affecting (as anxiety), adverse effect on the workplace functioning (as decrease in productivity), negative impact on health care quality (as patient safety), social consequences (on private and family life) and financial consequences (as the loss of income due to absences from work). Similarly, a meta-analysis on the effect of workplace discrimination due to race showed a negative impact on the attitude to work and on the physical and mental health of the affected persons [30].

Many of the above-mentioned consequences of workplace violence and discrimination obviously have a detrimental effect not only on an individual level, but also on a company level and on the societal level [31]. While longer sick leaves and absences from work may be the most important factors for the company, a loss of quality in health care has a serious impact on the society as a whole. Furthermore, as health professionals usually face high work demands, a loss of productivity in consequence of experiencing violence at work is a serious challenge in health care [31].

Most studies on workplace violence and discrimination against health care workers focus on nurses or physicians and come from North American and Asian countries. For Switzerland almost no evidence exists on this topic since hardly any studies were carried out on this serious and increasing problem in health care. An observational cross-sectional study from the French-speaking part of Switzerland found a very high prevalence of 84% of workplace violence among prehospital emergency care providers [22].

And finally, they are both very little studied and poorly explored in German-speaking countries and particularly in Switzerland with regard to the prevalence and health correlates of such discriminatory and violent or aggressive behaviors against and amongst health care workers.

For this reason, the present study has been conducted, mainly based on survey data collected in 2015/16 among hospital employees and particularly health professionals from German-speaking Switzerland and additionally compared with self-reported data from the Swiss Health Survey of 2017. The Swiss Health Survey is a cross-sectional data collection which is carried out every 5 years and collects data among the permanent resident population of Switzerland aged 15 years and over and living in private households.

Against the background of the lack of evidence in this regard, this study aimed to investigate the following research questions:

1. What is the prevalence of different and accumulated aspects or forms of violence and discrimination experienced at work among hospital employees and particularly among health professionals in Switzerland, and in comparison with the entire working population?
2. Is there consistently a pronounced and negative association and a halfway linear dose-response relationship between accumulated experiences and forms of workplace violence and/or discrimination on the one hand and different health outcomes on the other hand among hospital employees?

**Fig. 1:**
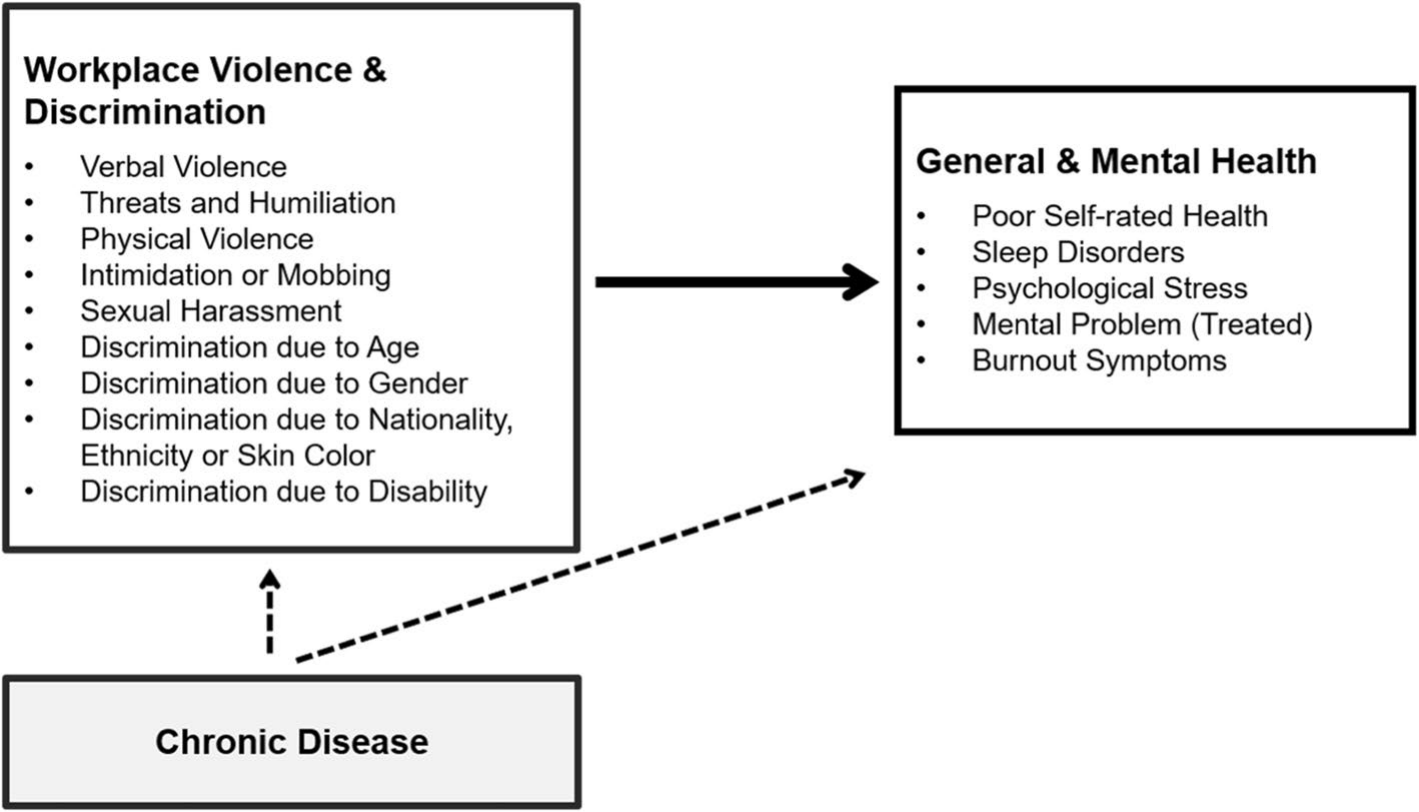
Theoretical path model showing the assumed and studied associations between exposure, outcome and confounding variables

Fig. 1 illustrates the theoretical path model of the postulated association between experienced workplace violence and/or discrimination and health status, which is assumed to be potentially confounded by chronic disease.

## Methods

### Data and study sample

This cross-sectional study is based on the data of a survey among the workforces of six public hospitals and rehabilitation clinics in German-speaking Switzerland collected in 2015 and 2016. The hospitals and clinics participating in the survey were selected by convenience sampling (non-probability sampling) and included one university hospital, one cantonal hospital, two district hospitals, and two rehabilitation clinics which are providing inpatient health care services.

All permanent employees of the selected hospitals were asked to participate in the survey anonymously and on a voluntary basis. Thus, there were no exclusion criteria or restrictions made concerning the different professions, hierarchical levels or hospital divisions and departments.

The self-administered paper-and-pencil questionnaire included 100 questions in total and took about 30 min to complete. Its overall aim was to gather information on the working conditions and health status of hospital employees and particularly health professionals.

Survey participants were allowed to fill out the questionnaire at work and during working time or at home if preferred. And they were asked to send the questionnaire back to the researchers by a prepaid reply envelope within 4 weeks, and got a reminder 1 week before submission deadline.

The size or workforces of the hospitals at the time of the data collection varied between 473 and 2175 permanent employees with a total staff of around 4450 employees (gross sample). In total, 1840 employees completed and returned the questionnaire (net sample). Thus, the overall participation or response rate was 41%, ranging from 36 to 49% between the participating hospitals and clinics.

This study was restricted to those 1567 employees who completed the questionnaire and were particularly asked about their stress feelings. In one of the six hospitals and clinics exactly and only this question about the perception of general psychological stress was unintentionally missing in the questionnaire which is why this hospital was excluded from the study.

In order to compare the study population of hospital employees with the general working population, additional and comparable survey data were needed. A nationally representative subsample of the Swiss Health Survey from 2017 was offered to be and used as a comparison group and standard or reference population. The two populations – the study population and the reference population – were largely comparable with regard to the time of measurement or data collection (2015/16 and 2017), employment status (working), age (working age), residential region (German-speaking Switzerland), and exposure assessment: Individuals from both populations were asked about their experiences of workplace violence and discrimination by exactly the same question or rather 9-item measure.

### Measures

#### Exposure variable(s)

##### Violence and discrimination at work

Experiences of workplace violence and discrimination were measured by the question “Have you experienced the following in the past 12 months at work?” with a note that multiple answers were possible. Ten answer categories were given: “discrimination due to age”, “discrimination due to gender”, “discrimination due to nationality, ethnicity or skin color”, “discrimination due to disability”, “verbal violence”, “threats and humiliation”, “physical violence”, “intimidation or mobbing”, “sexual harassment”, and “none of them”. The question and response options were adopted from the Swiss Health Survey, allowing to make a direct comparison with secondary survey data representing the working population of (German-speaking) Switzerland [32]. For the association analyses, a sum scale which simply adds up the number of the nine experienced and surveyed different aspects of workplace violence and/or discrimination was constructed. The sum scale starting with 0 and a possible maximum score of 9 was classified into three categories of “none” (0), “one” (1) and “accumulated” (2+).

#### Outcome variables

##### General and mental health

With the exception of burnout, which was assessed by an established multiple-item measure, single-item measures were utilized to assess the general and mental health status of the study population of hospital employees. Most of these widely used items were taken from the Swiss Health Survey, allowing to make a comparison with a representative sample of the working population in German-speaking Switzerland.

- *Self-rated health*. Self-reported general health status was measured by asking “How is your health status in general?” with response categories from 1 (“very good”) to 5 (“very bad”). Self-rated health (SRH) is an established and well-validated measure of general health, showing a strong association with both mortality [33] and morbidity [34]. Due to its strongly skewed distribution, SRH was dichotomized (and binary coded) into two categories, combining answers from “very good” and “good” (value 0) and from “moderate” to “very bad” (1), labeled as “poor SRH”, as suggested by the research literature [35].
- *Sleep disorders*. Sleeping problems were assessed by asking the respondents about having had complaints in the past 4 weeks such as difficulties in falling asleep or sleeping through, with three answer categories dichotomized into “none at all/only a little” (0) and “strong” (1), in order to calculate logistic regression analyses.
- *Psychological stress*. General stress in a psychological (and not physiological sense) was measured by a given definition (“Stress means a condition in which a person feels tense, restless, nervous or anxious or is unable to sleep at night because his/her mind is troubled all the time.”), followed by the question: “Did you feel stressed in the past 12 months?” For the analysis, response categories, initially on a five-point Likert Scale from 1 (“not at all”) to 5 (“very strong”), were then dichotomized and binary coded, distinguishing between 0 “less stressed” (“not at all”, “a little”, “moderate”) and 1 “strongly stressed” (“strong”, “very strong”). This single-item measure of general stress is a widely used and well-validated indicator of mental strain [36].
- *Mental problem*. In order to measure a psychological problem, survey participants were directly asked if they had been treated due to a mental problem in the past 12 months, with the answer options “no” (0) and “yes” (1).
- *Burnout*. Burnout symptoms were measured using the 6-item subscale of personal burnout from the Copenhagen Burnout Inventory (CBI) [37]. This personal burnout measure is only one of originally and totally three conceptualized burnout subscales of the CBI but the only one that was used in the German standard version of the Copenhagen Psychosocial Questionnaire (COPSOQ) from which the wording of the six items was taken. The six questions ask about the frequency of feeling tired, of being physically exhausted, of being emotionally exhausted, of thinking “I can’t go any longer”, of being drained, and of feeling weak and vulnerable to diseases. Each item can be answered on a five-point Likert scale ranging from 0 (“never”) to 4 (“always”). The sum score out of these answers of the CBI was calculated and ranged between 0 and 24, with values above 16 being considered as an increased risk of burnout.

#### Confounding variable

*Chronic disease* as a potential confounder was measured by the question “Do you have a chronic disease or health problem?” (yes/no), followed by the explanation that this is a condition which is already lasting or still ongoing for at least 6 months.

#### Control variables

Sex, age and education were used as control variables. Age was measured by asking about the age category the respondent belongs to (< 25 years, 25–34 years, 35–44 years, 45–54 years and ≥ 55 years). Education was measured by asking participants about their highest degree of education. The 12 given educational qualifications were categorized into four levels of education: 1 “low” (no vocational education), 2 “medium” (basic vocational education/apprenticeship), 3 “high” (higher vocational education or high-school diploma), and 4 “very high” (university degree).

For stratified analyses or rather differentiated descriptive statistics, study or survey participants were further categorized into four occupational groups (nurses and midwives, physicians and other academics, medical-therapeutic and medical-technical staff, administrative and other service staff).

### Analyses

To answer the first research question regarding the prevalence rates of workplace violence and discrimination among health care workers in German-speaking Switzerland, relative frequencies of exposure variables (single items and sum scale of different aspects of workplace violence and discrimination) were calculated for the entire study population and additionally stratified by the four occupational groups. Such descriptive statistics were provided for the study sample as well as for the comparable and representative subsample of the Swiss Health Survey of 2017 as a reference group, representing the working population of German-speaking Switzerland.

Multiple logistic regression analyses were then performed to study the second research question about the assumed and possibly confounded association and dose-response relationship between the accumulated number of experiences of violence and discrimination at work and different general and mental health outcomes. More precisely, multiple adjusted odds ratios (aOR) were calculated to estimate the relative and health-related risk of such experiences in the study population.

## Results

### Descriptive statistics

The prevalence of experiences of different forms or aspects of violence and discrimination at work among hospital employees (in the past 12 months) is shown in Table 1. With a look at the entirety of hospital employees, the most frequently reported form of discrimination was due to age (5%), followed by discrimination due to gender (4%), nationality, ethnicity or skin color (3%) and disability (less than 1%). In physicians and other academic staff, discrimination due to gender was the most prevalent form (8%).

**Table 1:** One-year prevalence of workplace violence and discrimination among occupational groups of hospital employees and compared with the working population of German-speaking Switzerland

	Nurses and midwives (*n* = 718)	Physicians and other academic staff (*n* = 293)	Medical-therapeutic and medical-technical staff (*n* = 221)	Administrative and other service staff (*n* = 325)	Total hospital employees (*N* = 1567)^a)^	Working population of German-speaking Switzerland (*N* = 8281)^b)^
	% (n)	% (n)	% (n)	% (n)	% (n)	% (n)^c)^
**Forms of violence**						
• Verbal abuse	9.3 (67)	6.8 (20)	3.6 (8)	4.6 (15)	7.1 (111)	4.7 (345)
• Threats and humiliation	6.1 (44)	5.1 (15)	4.5 (10)	4.0 (13)	5.2 (82)	4.1 (306)
• Physical violence	1.7 (12)	0.3 (1)	0.5 (1)	0.0 (0)	0.9 (14)	1.1 (75)
• Intimidation or mobbing	11.3 (81)	8.2 (24)	9.5 (21)	8.9 (29)	9.9 (155)	6.5 (473)
• Sexual harassment	1.5 (11)	0.7 (2)	0.0 (0)	0.6 (2)	1.0 (15)	0.8 (48)
**Forms of discrimination**						
• Due to age	4.5 (32)	4.4 (13)	4.1 (9)	6.2 (20)	4.7 (74)	5.7 (466)
• Due to gender	2.9 (21)	8.2 (24)	4.5 (10)	2.5 (8)	4.1 (64)	3.9 (313)
• Due to nationality, ethnicity or skin color	2.2 (16)	4.1 (12)	1.8 (4)	2.5 (8)	2.6 (41)	3.4 (233)
• Due to disability	0.3 (2)	0.0 (0)	0.9 (2)	0.6 (2)	0.4 (6)	0.8 (60)
**Total number of experienced forms of violence and/or discrimination**						
• 0 (none)	75.6 (543)	76.8 (225)	78.7 (174)	80.9 (263)	77.3 (1212)	81.7 (6870)
• 1 (one)	14.3 (103)	13.0 (38)	14.9 (33)	10.5 (34)	13.5 (211)	10.6 (823)
• 2+ (accumulated)	10.0 (72)	10.2 (30)	6.3 (14)	8.6 (28)	9.2 (144)	7.7 (566)

^a)^ The sum of the numbers of cases of all occupational groups (*N* = 1557) does not equal the total number of hospital employees (*N* = 1567) since 10 respondents did not provide information on their profession

^b)^ Subsample of the Swiss Health Survey 2017 consisting of all employed and surveyed persons aged 15 and older from the following Swiss cantons: BE, LU, UR, SZ, OW, NW, GL, ZG, SO, BS, BL, SH, AR, AI, SG, GR, AG, TG, ZH

^c)^ Relative frequencies (%) based on weighted (and extrapolated) data from the Swiss Health Survey 2017; number of cases (n) based on unweighted data

With regard to experiences of violence at work among the studied hospital employees, intimidation or mobbing was the most commonly reported form (10%), followed by verbal violence (7%), threats and humiliation (5%), sexual harassment (1%), and physical violence (1%). In the working population of German-speaking Switzerland, intimidation or mobbing was also the most frequently reported form of violence (7%). In comparison with other occupational groups, nurses and midwives were by far the most affected from all forms of violence.

Overall, almost a quarter (23%) of the surveyed hospital employees reported at least one form of discrimination or violence in the past year, whereby nurses and midwives were most frequently affected (24%), followed by physicians and other academic staff (23%), medical-therapeutic and medical-technical staff (21%) and administrative and other service staff (19%). Hospital employees and particularly health professionals were found to be more frequently affected by experiences of violence and discrimination at work than employed persons and working people in general, which make such experiences at work on average in “only” 18% of the cases.

### Multiple logistic regression analyses

Table 2 illustrates the associations between the experience of workplace violence and/or discrimination and different dimensions of health among hospital employees: After adjusting for sex, age, education (control variables) and chronic disease (potential confounding variable), experiencing one single form of discrimination or violence at work (compared to having not experienced any violence or discrimination) was significantly associated with strong sleep disorders (19% vs. 11%, aOR 2.0), strong psychological stress (19% vs. 11%, aOR 1.7) and increased burnout symptoms (14% vs. 6%, aOR 2.6). These associations were clearly more pronounced when having reported accumulated experiences of workplace violence and/or discrimination, i.e. more than one form. These most affected or exposed hospital employees show almost consistently – although not always significantly – the highest prevalence rate and relative risk for poor self-rated health (17%, aOR 1.6), strong sleep disorders (29%, aOR 3.1), strong psychological stress (33%, aOR 3.4), being treated for a mental problem (12%, aOR 1.4) or increased burnout symptoms (21%, aOR 4.1), compared to those who have not made any of such experiences at all. The initially clear and statistically significant dose-response relationship or gradient found for poor self-rated health in the simple model turned into a non-significant association and a non-linear relationship in the extended model including the potentially confounding variable of chronic disease. Only for having a mental problem, prevalence rates and adjusted odds ratios as proxies for the relative risk were not significantly increased for the most affected and exposed from the very beginning and in both models.

**Table 2:** Multiple-adjusted and marginally confounded associations between the total number of experiences of workplace violence and/or discrimination and several health outcomes among hospital employees (*N* = 1567)

	Poor self-rated health			Strong sleep disorders			Strong psychological stress			Being treated for mental problem			Increased burnout symptoms		
	%	aOR^1)^	95% CI	%	aOR^1)^	95% CI	%	aOR^1)^	95% CI	%	aOR^1)^	95% CI	%	aOR^1)^	95% CI
**Total study population**	**11.1**			**13.3**			**14.3**			**7.5**			**8.4**		
MODEL 1 (simple)															
**Experienced forms of workplace violence and/or discrimination**															
• 0 (*n* = 1204)	9.6	1		10.6	1		11.3	1		7.3	1		5.8	1	
• 1 (*n* = 211)	15.2	1.68*	1.10–2.56	18.6	1.92***	1.30–2.85	18.9	1.82**	1.22–2.71	5.8	0.79	0.42–1.46	14.4	2.71***	1.72–4.28
• 2+ (*n* = 144)	17.4	1.97**	1.23–3.16	28.5	3.36***	2.24–5.04	33.1	3.86***	2.60–5.75	11.8	1.71	0.98–2.96	21.1	4.33***	2.70–6.93
No. of cases in model		1559			1552			1507			1548			1535	
MODEL 2 (extended)															
**Experienced forms of workplace violence and/or discrimination**															
• 0 (*n* = 1204)	9.6	1		10.6	1		11.3	1		7.3	1		5.8	1	
• 1 (*n* = 211)	15.2	1.59	1.00–2.54	18.6	1.97***	1.31–2.95	18.9	1.74**	1.15–2.62	5.8	0.68	0.36–1.31	14.4	2.55***	1.60–4.07
• 2+ (*n* = 144)	17.4	1.57	0.93–2.64	28.5	3.11***	2.03–4.74	33.1	3.37***	2.23–5.08	11.8	1.44	0.81–2.56	21.1	4.11***	2.53–6.66
**Chronic disease**															
• No (*n* = 1142)	4.4	1		10.5	1		11.5	1		5.7	1		6.9	1	
• Yes (*n* = 409)	29.8	9.54***	6.62–13.74	21.2	1.98***	1.44–2.72	22.8	2.05***	1.50–2.80	12.3	2.29***	1.54–3.40	12.7	1.87**	1.26–2.75
No. of cases in model		1515			1509			1462			1505			1493	

*p < 0.05, **p < 0.01, ***p < 0.001

^1)^ Odds ratios adjusted for sex, age and education (control variables)

In other words, a strong association and clear and stable dose-response relationship was observed between the number of experiences of workplace violence and/or discrimination and three of the five studied health outcomes. And this relationship was not substantially confounded by chronic disease (extended model) which in turn was found to be a strong and significant risk factor of poor general and mental health outcomes itself.

## Discussion

This study aimed to estimate and investigate the prevalence rates and health correlates of different and accumulated forms of workplace violence and discrimination experienced by hospital employees in German-speaking Switzerland. This is a long identified and globally recognized problem and a frequently observed phenomenon in health care settings and occupations which, nevertheless, is still largely underreported and unexplored in some countries, particularly in Switzerland.

As regards the frequency of workplace violence and discrimination among hospital employees and particular health professionals almost every fourth (23%) of the study population reported at least one form of experienced discrimination or violence at work in the past 12 months, compared to only every sixth (18%) in the entire working population of German-speaking Switzerland. With a view to the occupational groups, nurses and midwives were most often affected by violence at work, whereas physicians and other academics were most often affected by discrimination due to nationality, ethnicity or skin color and particularly due to gender. The most frequent form of violence experienced by hospital employees was intimidation or mobbing (10%), whereas ageism was the most commonly reported type of discrimination (5%).

Hence, the finding of a comparably high prevalence of workplace violence and discrimination in hospital employees compared with the general working population is in accordance with previous research, which has shown that health care workers are at special risk for workplace violence, as they work with people who are in distress [19]. Working in direct patient contact means to be faced with people whose behavior can be affected by acute illness and pain, psychiatric and neurological disorders, intoxications and substance abuse [19, 38]. Nurses are at particular risk, as this is usually the professional group which is longest and most frequently in contact with and therefore exposed to patients [38]. There is also evidence that certain hospital units are more confronted with violence from patients or relatives, such as emergency and psychiatric wards [17, 22, 39, 40]). Intimidation or mobbing in this study was found to be the most commonly reported form of workplace violence and twice as often than in another study conducted in nursing homes in Switzerland, which found a prevalence of mobbing in the past 6 months of nearly 5% among care workers [41]. Regarding ageism, earlier studies showed that discrimination on the grounds of being “too young” is at least as common as on the grounds of being “too old” [42]. Although employees of these two age groups are confronted with different prejudices and potential occupational disadvantages, there is evidence that ageism is associated with a lower level of affective commitment in both of them [42]. Another finding of this study is that gender discrimination is most commonly reported among physicians and other academic staff. A possible explanation might be that this form of discrimination is becoming increasingly important in employees with higher education. Equal rights for women and men and gender equality is an important concern in politics and policies since many years in Switzerland [43]. However, with a look at the Gender Monitoring Report from Swiss University faculties of medicine in 2014, there is still a considerable gender gap in higher positions: While over 50% of medical graduates with a master’s degree are women, the proportion drops to 10% on full professor level [44].

With a look at the one-year prevalence of violence expected by health personal in an international comparison, a very broad range can be observed, ranging from 3% (Portugal) to 17% (South Africa) for physical attacks, from 17% (Portugal) to 67% (Austria) for verbal violence and from 11% (Australia) to 31% (Bulgaria) for mobbing [19]. One reason for these large differences between countries might be a limited comparability of the underlying studies, for example in relation to the methodology (study design, definitions used), setting (differences in health care systems, hospital versus outpatient sector, medical specialties), sample (personal characteristics of the study population) and cultural peculiarities (including differences in awareness and reporting systems).

Besides increased prevalence rates of specific forms of violence and/or discrimination at work and/or among particular occupational groups, accumulated experiences of workplace violence and/or discrimination among hospital employees and particularly health professionals were found to be strongly associated with poor mental health outcomes such as strong sleep disorders, strong stress feelings or increased burnout symptoms. The prevalence (or relative frequency) and the odds or likelihood (or relative risk) of these poor health outcomes were shown to gradually, substantially and significantly increase with the self-reported number of experiences (0, 1, 2+) of violence and/or discrimination made at work, suggesting a dose-response relationship. The strength of association and the clear dose-response relationship across most studied outcomes are a good indication for a causal relationship.

This finding is in line with a series of earlier international studies and findings and particularly supported by a systematic literature review published in 2021 on the consequences of workplace violence among employees working in the human service industries (health care, social care and education), showing a significant association with psychological problems in 19 out of 24 prospective studies [45]. These studies which investigated the effect of physical or psychological violence as predictors of mental health outcomes reported mainly burnout symptoms, symptoms of psychological distress or posttraumatic stress disorder (such as intrusion, negative changes in cognition or mood, changes in arousal and reactivity), anxiety or depressive symptoms and sleep disorders [45]. A previous study among Swiss nursing home care workers found a remarkably higher odds of health complaints in those directly affected by mobbing [41]. Another study on the consequences of workplace violence among Chinese physicians showed a positive correlation with psychological stress, and a negative association with subjective sleep quality and subjective health [46]. Partly in contrast to this Chinese study, we did not find a significant association of experienced violence or discrimination at work with self-reported health, at least not after adjusting for chronic disease. In other words: Although prevalence rates of poor self-rated health were significantly increased among hospital employees who experience and report at least one form of violence and/or discrimination at work, this was mainly due to their higher proportion of chronically diseased who in turn showed an almost tenfold higher risk of being in poor general and self-reported health than those without a chronic disease. Regarding the other studied poor health outcomes, chronic disease not turned out to be an important confounder, as having a chronic disease only about doubled (and not tenfold increased) the risk of having strong sleep disorders, feeling strong psychological stress, being treated for a mental problem or showing increased burnout symptoms. For these health measures, chronic disease only slightly explained and therefore reduced the strong association found between workplace violence or discrimination and poor health outcomes. But in case of poor self-rated health, a gradually and significantly increased risk with the increasing number of experiences of violence and discrimination at work turned out to be not statistically significant anymore when the association was adjusted for chronic disease.

In sum – it is not really surprising but at the same time has not been shown before at least for health care workers in Switzerland – we found that accumulated experiences of violence and discrimination at work are a strong stressor and risk factor for sleep disorders, psychological stress and burnout, even though it does not seem to cause severe mental problems.

Burnout symptoms in health care workers are very common. A meta-analysis among medical and surgical residents estimates its global prevalence to be around 51% [47]. Burnout is a psychological syndrome, which can be regarded as a prolonged result to chronic stressors at work, consisting of three different domains, namely emotional exhaustion, depersonalization and low personal achievement [48]. It seems to be more prevalent in professions comprising an intensive work with other people, such as care giving or teaching roles [48]. As burnout in health care staff is associated with higher costs and major medical errors [49], studies investigating its risk factors are numerous. Previous studies suggest that mistreatment of health care professionals (including harassment, bullying, discrimination and physical violence) contribute to the development of burnout symptoms in this population [50–52]. However, there is a broad range of other factors related to health care organizations and systems as well as individual characteristics knowing to play an important role in the development of or protection from burnout symptoms [53].

### Limitations

This study has some limitations that have to be considered with regard to the study results: First, the study is based on cross-sectional data, which do not allow to test for causality. Moreover, reverse causality cannot be excluded either. For instance, a high level of stress can trigger unsocial behavior towards colleagues or impatient behavior towards patients, which themselves could increase the risk of discriminating statements from colleagues or aggressive behavior in patients. This raises the question whether psychological stress is a consequence of discrimination or violence experiences or if a high stress level could also be the starting point of a vicious circle ending in violence or discrimination experiences.

Secondly, the question measuring experiences of workplace violence or discrimination did not distinguish between internal and external violence and did not assess the true extent of the experiences, which would have been helpful for a more accurate estimation of the exposure or strain and for the interpretation of the results. Additionally, there is a risk for potential recall bias, as the question on experienced violence and discrimination refers to a period of 12 months before completion of the questionnaire.

In view of the described overall response rate of around 40%, there is a risk for potential selection bias (non-response bias). Also, as participants were allowed to complete the questionnaire during working time, a potential response bias should be taken into account, as people could have answered in a way they considered to be desirable by their company.

The internal validity of the study findings seems to be fairly high as strong associations and clear dose-response relationships were almost consistently found for the studied relationship between the number of different forms of experienced workplace violence and discrimination and various physical and mental health outcomes and as associations were adjusted for several control and potentially confounding variables. However, the external validity of the findings is limited since a non-random sampling (so-called convenience sampling) method was used to select the survey participants on a company level. This and the small number of selected or included hospitals and clinics and the self-selection and rather low participation of employees that were taking part in the survey may lead to non-representative and systematically biased results, particularly as regards the size and type of the companies or hospitals and clinics and the working and health conditions of their workforces or employees. In other words: The generalizability of the study findings is questioned. But this limits possibly the generalizability of the prevalence of individual or cumulated forms and events of workplace violence and discrimination in the study population and among the studied health professions and not necessarily the found strong associations of such cumulated negative experiences and social conflicts at work with the studied health outcomes.

This non-random sampling to recruit survey participants (hospitals and clinics) could affect the study results insofar as the “true” prevalence of workplace violence and discrimination might be underestimated since companies and health care institutions in which many such events of violence and discrimination between employees and coworkers or patients are taking place may tend to be less willing to participate in a survey on “work and health” (on a company level) for fear of poor results. Hospital employees who work in a rather bad or conflictive work atmosphere and experience frequently violence and discrimination at work likewise are possibly less ready to participate in such a survey (on an individual level) as they may anticipate a frustrating lack of consequences or improvement as a result of the survey findings.

## Conclusions

The present study could shed some light on a serious and growing problem and a blind spot in health (services) research in Switzerland. The comparably high prevalence of workplace violence and discrimination among hospital staffs and particularly among health professionals and its significant association with sleep disorders, stress feelings and burnout symptoms underline the importance of an active combat against these undesired but still common behaviors in health care. Strategies to prevent violence should primarily focus on nurses and midwives, which have been identified as the most affected professional group of hospital employees in this study.

### Implications for managers and decision-makers

Managing violence and discrimination at workplace is an important issue that has been addressed by different intervention studies in the health care setting [54–58]. More or less effective interventions were identified such as training sessions and education programs (for example in communication skills) or organizational policies and practicies (such as introducing reporting systems or safety procedures, formulating no-tolerance policies, promoting people-oriented culture through open communication, cooperation, trust and participation in decision-making).

And it is a challenging task not only for clinical practice, but also for health policy, requiring a holistic approach according to the complexity of these phenomenon. Therefore, prevention and dealing strategies in hospitals should not only focus on organizational factors, but also on the level of the individual employees and their interactions. With regard to the latter, previous studies suggest to provide training programs for health care workers, for example in communication skills [54]. With regard to organizational factors in hospitals, the development of a no-tolerance policy or environmental changes improving the safety (e.g. the presence of security systems) are some examples which are assumed to limit workplace violence [38]. However, another important issue is the way how to handle discrimination or violence experiences that have already taken place, in a way that minimizes its negative consequences on the victims. For example, Schat and Kelloway found that social support from colleagues and supervisors is able to buffer the negative consequence of workplace violence/aggression on physical and psychological health as well as working attitudes in health care employees [59].

## Data Availability

Individual data were collected by random and full sample surveys among the workforces of several public hospitals and rehabilitation clinics. Data were collected anonymously and on a voluntary basis. However, data are not publicly accessible and freely available since the use and analysis of the pooled data and the publication of any research findings and study results out of it are restricted by contract with the participating companies (hospitals, clinics) to the University of Zurich (Epidemiology, Biostatistics and Prevention Institute) and the collaborating Careum Research, a division of the Careum Foundation. As contracted, the use of the data is basically limited to the two research institutions and disclosure and delivery of the data therefore is not permitted. In order to get an exceptional permission and possible conditional access to the survey data for scientific purposes the corresponding author as the principal investigator and the responsible for the data collection needs to be contacted.

## Abbreviations

aOR: Adjusted odds ratio
CBI: Copenhagen Burnout Inventory
CI: Confidence interval
COPSOQ: Copenhagen Psychosocial Questionnaire
FOPH: Federal Office of Public Health
ILO: International Labour Organization
SECO: State Secretariat for Economic Affairs
SRH: Self-rated health
SUVA: Swiss National Accident Insurance Fund
